# Skeleton-guided 3D convolutional neural network for tubular structure segmentation

**DOI:** 10.1007/s11548-024-03215-x

**Published:** 2024-09-12

**Authors:** Ruiyun Zhu, Masahiro Oda, Yuichiro Hayashi, Takayuki Kitasaka, Kazunari Misawa, Michitaka Fujiwara, Kensaku Mori

**Affiliations:** 1 Graduate School of Informatics, Nagoya University, Furo-cho, Chikusa-ku, Nagoya, Aichi Japan; 2 Information Strategy Office, Information and Communications, Nagoya University, Furo-cho, Chikusa-ku, Nagoya, Aichi Japan; 3 Information Technology Center, Nagoya University, Furo-cho, Chikusa-ku, Nagoya, Aichi Japan; 4 School of Information Science, Aichi Institute of Technology, 1247 Yachigusa, Yakusa-cho, Toyota, Aichi Japan; 5 Aichi Cancer Center Hospital, 1-1 Kanokoden, Chikusa-ku, Nagoya, Aichi Japan; 6 Nagoya University Graduate School of Medicine, 65 Tsurumai-cho, Showa-ku, Nagoya, Aichi Japan

**Keywords:** 3D convolutional network, Tubular structure segmentation, CT image

## Abstract

**Purpose:**

Accurate segmentation of tubular structures is crucial for clinical diagnosis and treatment but is challenging due to their complex branching structures and volume imbalance. The purpose of this study is to propose a 3D deep learning network that incorporates skeleton information to enhance segmentation accuracy in these tubular structures.

**Methods:**

Our approach employs a 3D convolutional network to extract 3D tubular structures from medical images such as CT volumetric images. We introduce a skeleton-guided module that operates on extracted features to capture and preserve the skeleton information in the segmentation results. Additionally, to effectively train our deep model in leveraging skeleton information, we propose a sigmoid-adaptive Tversky loss function which is specifically designed for skeleton segmentation.

**Results:**

We conducted experiments on two distinct 3D medical image datasets. The first dataset consisted of 90 cases of chest CT volumetric images, while the second dataset comprised 35 cases of abdominal CT volumetric images. Comparative analysis with previous segmentation approaches demonstrated the superior performance of our method. For the airway segmentation task, our method achieved an average tree length rate of 93.0%, a branch detection rate of 91.5%, and a precision rate of 90.0%. In the case of abdominal artery segmentation, our method attained an average precision rate of 97.7%, a recall rate of 91.7%, and an F-measure of 94.6%.

**Conclusion:**

We present a skeleton-guided 3D convolutional network to segment tubular structures from 3D medical images. Our skeleton-guided 3D convolutional network could effectively segment small tubular structures, outperforming previous methods.

## Introduction

Three-dimensional (3D) tubular structure information is critical for clinical practice. For example, the blood vessel information could be used for blood flow simulations for surgical assistance. Moreover, segmenting tubular structure organs from CT volumetric images, such as the bronchus, is widely used in diagnosing and treating lung diseases. Compared to general organ segmentation tasks, correctly segmenting 3D tubular structures is challenging due to the variation in volume and branching patterns. Therefore, developing an effective automated method for segmenting 3D tubular structures is imperative.

Over the last decade, deep learning techniques have been widely utilized in medical image analysis. Various 3D deep models have been introduced for tubular structure segmentation. Wang et al. [1] proposed a spatial context-aware fully connected network for 3D tubular structure segmentation. They introduced 3D recurrent convolutional layers, which leveraged spatial information from features in an encoder–decoder architecture. Their method achieved an 88.7% Dice score over a bronchus dataset. However, thick bronchi were under-segmented in their results. Yu et al. [2] employed the residual block structure in the U-Net and fused multi-resolution feature maps to improve segmentation accuracy. Their method achieved 71.7% and 76.5% Dice scores in hepatic veins and portal veins segmentation, respectively. However, several distal blood vessels were not segmented well in their results.

Compared to the general organ segmentation task, 3D tubular structure segmentation presents challenges related to the volume imbalance issue. Specifically, in medical images, the tubular structure such as airways consists of a main branch and distal branches. The distal branches are usually much smaller than the main branch. Precisely segmenting the distal branches is difficult in tubular structure segmentation. Shit et al. [3] presented a topology-preserving loss to segment tubular structures. They proposed a connectivity-preserving metric to evaluate tubular and linear structure segmentation based on intersecting skeletons with ground truth. The metric is used as a loss function during the deep model training stage. Their method achieved an 87.8% Dice score over a 3D brain vessel dataset. However, their method tended to cause over-segmentation of blood vessels, leading to false positive connections. The approach proposed by Qin et al. [4] tackled tubular structure segmentation by transforming the conventional segmentation task into 26 neighbor voxel-wise connectivity prediction tasks. This approach achieved a Dice score of 90.2% on a bronchus dataset, indicating its effectiveness in accurately segmenting tubular structures. These results emphasize the importance of topological information in tubular structure segmentation.

For the tubular structure, the skeleton information is crucial to precisely demonstrate complicated branch patterns of the structure. The motivation of our work is to further explore the use of skeleton information of tubular structures and improve segmentation accuracy. To this end, we present a skeleton-guided 3D convolutional neural network. Moreover, we present a sigmoid-adaptive Tversky loss (STL) to train our deep model for segmenting the skeleton of the tubular structure. Our loss achieves a voxel-wise focal function in that each voxel is adaptively distributed with a different gradient ratio according to the corresponding predicted likelihood. The contributions in this paper are summarized as follows:

1. We introduce a novel skeleton-guided 3D convolutional neural network to improve segmentation performance on tubular structures by leveraging skeleton information.
2. We propose an innovative loss function that focuses on segmenting the skeleton of tubular structures.

## Methods

**Fig. 1:**
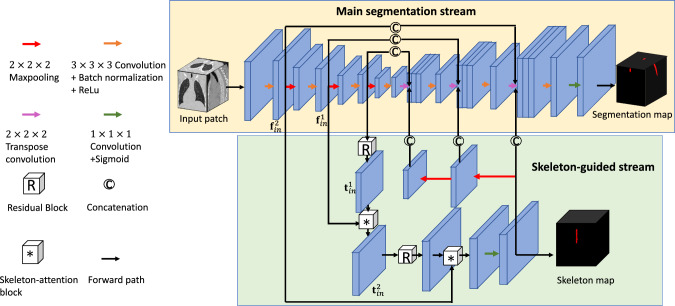
The network architecture of our SG-CNN. The yellow region presents the main segmentation stream, and the green region presents the skeleton-guided stream. Feature maps in main segmentation stream are fed into the skeleton-guided stream for skeleton extraction. Extracted skeleton feature maps are fed back to main segmentation stream to strengthen tubular structure segmentation

### Skeleton-guided convolutional neural network (SG-CNN)

We first introduce the architecture of our skeleton-guided convolutional neural network (SG-CNN) as shown in Fig. 1. The SG-CNN has two streams: the main segmentation stream and the skeleton-guided stream. The main segmentation stream is used for tubular structure segmentation, and the skeleton-guided stream is specifically designed for extracting the skeleton map of the tubular structure.

**Fig. 2:**
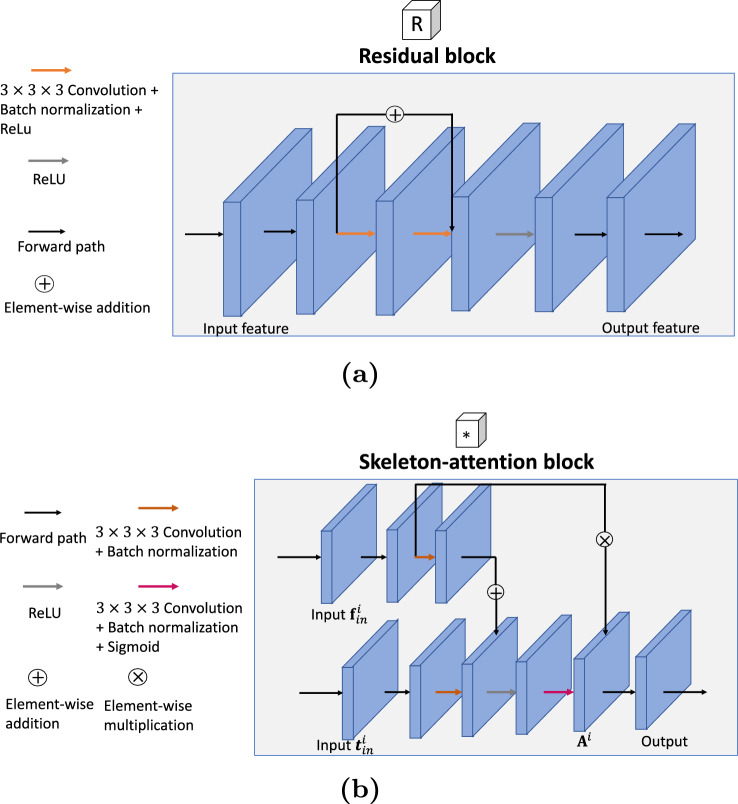
Details of the residual block and the skeleton-attention block in our SG-CNN. **a** residual block **b** skeleton-attention block

#### Main segmentation stream

The main segmentation stream refers to the generic encoder–decoder architecture [5] with skip connections. The encoder module is composed of three downsampling blocks, each containing two $3 \times 3 \times 3$ kernel of convolutional layers, followed by a max pooling layer. The decoder module symmetrically has three upsampling blocks in respect to the encoder module. Each upsampling block includes two convolutional layers with $3 \times 3 \times 3$ kernels, followed by a transpose convolutional layer with a $2 \times 2 \times 2$ kernel for upsampling, and concatenation with corresponding feature maps from the encoder module and the skeleton-guided stream. Finally, a convolutional layer with a $1 \times 1 \times 1$ kernel and a sigmoid layer is utilized to predict segmentation likelihood maps.

#### Skeleton-guided stream

The skeleton-guided stream comprises two skeleton-guided blocks corresponding to the feature maps generated by the encoder module. Each skeleton-guided block has a residual block, followed by a skeleton-attention block.

The residual block consists of two $3 \times 3 \times 3$ kernel-based convolutional layers with a skip connection, as shown in Fig. 2a. Firstly, the high-level feature map of the encoder module is fed into the residual block to enhance feature representations. The skeleton-attention block necessitates a pair of inputs, derived from the corresponding feature maps of both the encoder module and the preceding residual block.

**Fig. 3:**
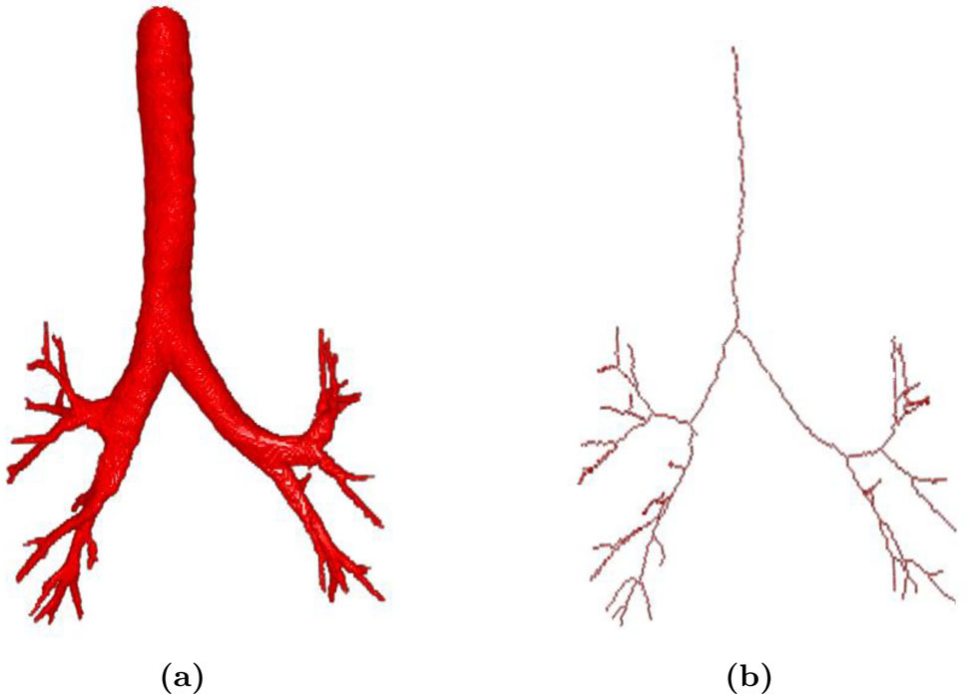
An example of the airway ground truth and the corresponding skeleton. **a** airway ground truth. **b** the corresponding skeleton ground truth. The skeleton ground truth is calculated by applying a skeletonization algorithm [7] to the airway ground truth

The skeleton-attention block guides to extract skeleton information of tubular structures via an attention mechanism, as shown in Fig. 2b. Let $\textbf{t}_\textrm{in}^i$ and $\textbf{f}_\textrm{in}^i$ denote the input feature maps from the skeleton-guided stream and the main segmentation stream at the $i-$th skeleton-attention block, respectively. We first calculate an attention map $\textbf{A}^i$ that is written as

1 $$\begin{aligned} \textbf{A}^i = \sigma (\textrm{ReLU}(\textrm{Conv}_{1 \times 1 \times 1}(\textbf{t}_\textrm{in}^i) + \textrm{Conv}_{1 \times 1 \times 1}(\textbf{f}_\textrm{in}^i))). \end{aligned}$$

where $\textrm{Conv}_{1 \times 1 \times 1}$ represents $1 \times 1 \times 1$ kernel convolutional layer. $\sigma$ and $\textrm{ReLU}$ represent a rectified linear unit (ReLU) and a sigmoid function $\sigma (\cdot )$, respectively. Note here that our attention map is acquired by leveraging two input feature maps, which is different from the self-attention mechanism [6]. Subsequently, the attention map $\textbf{A}^i$ is multiplied by the input $\textbf{t}_\textrm{in}^i$ to obtain an attention-guided feature map, and the map then propagates to the next skeleton-guided block for extracting skeleton information from additional feature maps. The prediction of the skeleton-guided stream is acquired similarly to the main segmentation stream. The extracted skeleton features were fed back to the main segmentation stream through concatenation. We employ a skeletonization algorithm [7] to tubular structure ground truth to acquire binary skeleton ground truth. For 3D binary skeleton ground truth, we use 6-adjacency for the foreground and 26-adjacency for the background. An example of the skeleton ground truth is shown in Fig. 3.

### Loss function for skeleton segmentation

In a skeleton ground truth map, the foreground voxels are much fewer than the background voxels, which might cause the gradient imbalance issue [8]. Given a foreground voxel and a background voxel, the gradient ratio represents the ratio of the foreground gradient’s magnitude to the background gradient’s magnitude. If a foreground voxel is surrounded by background voxels and the gradient ratio is small, the foreground gradients might be eroded by the surrounding background gradients, which denotes the gradient imbalance issue [8]. Our proposed sigmoid-adaptive Tversky loss function (STLoss) aims to mitigate the gradient imbalance issue and improve segmentation performance on skeletons.

#### Gradient imbalance

The gradient imbalance could sensibly impact the segmentation performance. In this paper, we use the Dice loss [9] to analyze the gradient imbalance of a prediction map. The Dice loss is written as

2 $$\begin{aligned} L_D (\textbf{p}, \textbf{g}) = 1 - \frac{2\sum _{i=1}^{N}p_ig_i}{\sum _{i=1}^{N}p_i + \sum _{i=1}^{N}g_i}, \end{aligned}$$

where $p_i \in \textbf{p}$ and $g_i \in \textbf{g}$ denote the predicted likelihood result and the ground truth at the $i-$th voxel. $p_i$ is in the range (0, 1), and $g_i$ takes a binary value of 0 or 1. The voxel index is represented by $i\in [1, N]$. This loss assigns the same foreground gradient to each foreground voxel, denoted as $p_f$, and the same background gradient to each background voxel denoted as $p_b$. The gradient ratio of the foreground voxel to the background voxel is

3 $$\begin{aligned} R_{D} (\textbf{p}, \textbf{g})&= \left|\frac{\partial L_D}{\partial p_f} \Big / \frac{\partial L_D}{\partial p_b}\right|\nonumber \\  &= \left|\frac{-\left( \left( \sum _{i=1}^{N}p_i + \sum _{i=1}^{N}g_i\right) -\sum _{i=1}^{N}p_ig_i\right) }{\sum _{i=1}^{N}p_ig_i}\right| \nonumber \\&= \frac{2}{1 - L_D (\textbf{p}, \textbf{g})} - 1. \end{aligned}$$

Equation (3) indicates that the gradient ratio $R_{D}$ is proportion to the Dice loss. When the overall Dice loss of a segmentation map is relatively small, the corresponding gradient ratio $R_D$ tends to be small. This scenario can lead to a gradient erosion problem. Consequently, the training process may struggle to classify foreground voxels surrounded by background voxels.

#### Sigmoid-adaptive Tversky loss (STL)

As aforementioned, the gradient ratio tends to be small as the Dice loss decreases. Consequently, it is difficult for the network to segment foreground voxels surrounded by background voxels.

The Tversky loss [10] mitigates the gradient imbalance issue by adding hyper-parameters $\alpha$ and $\beta$. The Tversky loss could be written as

4 $$\begin{aligned} L_T (\textbf{p}, \textbf{g}) = 1 - \frac{\sum _{i=1}^{N}p_ig_i}{\sum _{i=1}^{N}\alpha p_i + \sum _{i=1}^{N}\beta g_i}, \end{aligned}$$

where $\alpha +\beta =1$. Similarly to the Dice loss, we could calculate the gradient ratio of the Tversky loss is

5 $$\begin{aligned} R_{T} (\textbf{p}, \textbf{g}) = \left|\frac{\partial L_T}{\partial p_f} \Big / \frac{\partial L_T}{\partial p_b}\right| =\frac{1}{\alpha }\cdot \frac{1}{1 - L_T (\textbf{p}, \textbf{g})} - 1. \end{aligned}$$

We observe that the gradient ratio in the Tversky loss is influenced not only by the overall loss but also by the hyper-parameter $\alpha$. However, relying on adjusting hyper-parameters cannot adaptively modify the gradient ratio for every voxel. The final prediction results could be dilated if the gradient ratio is too large [8].

**Fig. 4:**
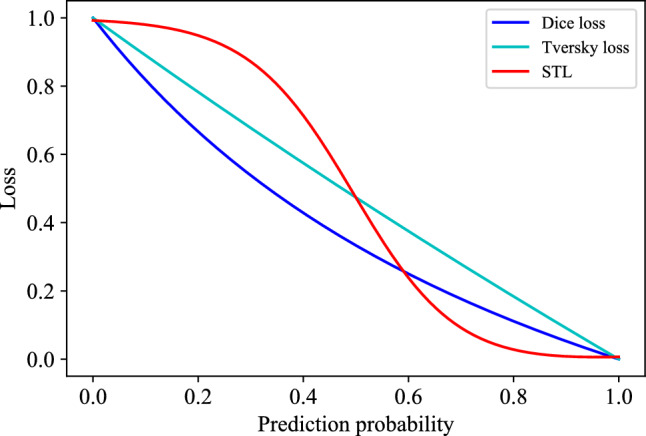
The comparison of losses. For the Tversky loss, the $\alpha$ and $\beta$ were set to 0.1 and 0.9, respectively. For our STL, the $\alpha$, $\beta$, and $\gamma$ were set to 0.1, 0.9, and 10, respectively. We noted that our STL exhibits more intense variability compared to other losses when the prediction probability approaches 0.5

We expect a loss function that could adaptively adjust the gradient ratio in a voxel-wise manner. To this end, we propose our sigmoid-adaptive Tversky loss (STL) which could voxel-wisely adjust the gradient ratio. Our loss function focuses on foreground voxels and background voxels with low prediction confidence, i.e., the prediction likelihood is close to 0.5. These voxels are generally hard to segment. The STL achieves a voxel-wise focal function by integrating a modified sigmoid function,

6 $$\begin{aligned} L_{ST} (\textbf{p}, \textbf{g}) = 1 - \frac{\sum _{i=1}^{N}\sigma (\gamma (p_i-0.5))g_i}{\sum _{i=1}^{N}\alpha p_i + \sum _{i=1}^{N}\beta g_i}, \end{aligned}$$

where $\sigma (\cdot )$ represents the sigmoid function. $\alpha$ and $\beta$ are two hyper-parameters that are similar to those of the Tversky loss, and $\alpha + \beta =1$. $\gamma$ is the third hyper-parameter and the $\gamma (p_i - 0.5)$ term in Eq. (6) mapping the $\sigma (\gamma (p_i-0.5))$ term to the value range (0, 1). A larger $\gamma$ causes a larger gradient magnitude when $p_i$ closes to 0.5. The gradient ratio of our STL loss is calculated by,

7 $$\begin{aligned} R_{ST} (\textbf{p}, \textbf{g})&= \left|\frac{\partial L_{ST}}{\partial p_f} \Big / \frac{\partial L_{ST}}{\partial p_b}\right| \nonumber \\&\quad =\frac{\gamma \sigma (\gamma (p_i{-}0.5))(1{-}\sigma (\gamma (p_i-0.5)))}{\alpha }\nonumber \\&\quad \cdot \frac{1}{1 {-} L_{ST}(\textbf{p}, \textbf{g})}{-} 1, \end{aligned}$$

where $p_i$ is the prediction likelihood of the $i-$th voxel. In our STL, voxels are adaptively assigned a gradient ratio according to the prediction likelihood $p_i$. Our loss function is different from the general class-wise weighting loss function with a voxel-wise adaption manner. Foreground voxels with low prediction confidence have a large gradient ratio, encouraging the network to learn from and improve the segmentation performance. An intuitive comparison of different losses is shown in Fig. 4. The hyper-parameters were empirically selected. We could observe that the loss variation trend of our STL is more pronounced compared to the other two losses when the prediction probability approaches 0.5. This suggests that the voxel with low prediction confidence is assigned a larger gradient magnitude compared to the voxel with high prediction confidence, achieving a voxel-wise gradient ratio adaptation.

**Table 1:** The acquisition parameters of datasets

Dataset	Slice size (pixel)	Slice spacing (mm)	Patch size (pixel)
Airway	$512\times 512$	0.5$-$1.0	$128\times 128\times 64$
Abdominal artery	$512\times 512$	0.5	$64\times 64\times 64$

In our overall loss function, we utilize the Dice loss for tubular structure segmentation, denoted as $L_\textrm{seg}$, and the STL for the loss function of the skeleton structure segmentation, denoted as $L_\textrm{skel}$. The overall loss function is

8 $$\begin{aligned} {L_\textrm{overall} (\textbf{p}, \textbf{g}, {\hat{\textbf{p}}}, {\hat{\textbf{g}}})} =\,&{L_\textrm{seg} (\textbf{p}, \textbf{g})} + {L_\textrm{skel} ({\hat{\textbf{p}}}, {\hat{\textbf{g}}})}\nonumber \\ =\,&{L_{D} (\textbf{p}, \textbf{g})} + {L_{ST} ({\hat{\textbf{p}}}, {\hat{\textbf{g}}})}, \end{aligned}$$

where $\textbf{p}$ and $\textbf{g}$ represent the segmentation likelihood maps of the tubular structure and the corresponding ground truth, respectively. ${\hat{\textbf{p}}}$ and ${\hat{\textbf{g}}}$ represent the segmentation likelihood maps of the skeleton and the corresponding ground truth, respectively.

## Experiments and results

### Experimental settings

We evaluated our method on two challenge datasets for different tubular structure segmentation tasks including the airway segmentation task and abdominal artery segmentation task. The airway segmentation dataset was a public dataset acquired from Shanghai Jiao Tong University [11]. The dataset consists of 90 cases, comprising 70 cases obtained from the LIDC dataset [12] and 20 cases obtained from the training set of the EXACT’09 [13]. We randomly selected 80 cases and generated about 7000 patches for training. The remaining 10 cases were used for testing. The abdominal artery dataset was a private dataset containing 35 arterial phase-contrasted abdominal CT volumetric images for abdominal artery segmentation. The volumetric images were produced using the Siemens SOMATOM Force CT scanner and were obtained from the Aichi Cancer Center Hospital. The ground truth artery volumes were manually annotated by engineering researchers and confirmed by a surgeon from the Aichi Cancer Center Hospital. We revised the manuscript by adding details about our private data. We used about 12,000 patches generated from 30 cases for training, and the left five cases for testing. All of the training cases were normalized to [0, 1]. No data augmentation was employed. The acquisition parameters of both datasets are shown in Table 1.

For the evaluation metrics, we employed distinct metrics for two different segmentation tasks. We employed three evaluation metrics for airway segmentation: tree length detected rate (TR), branch detection rate (BD), and precision rate. The airway ground truth is divided into several branches using bifurcation points. We first find the bifurcation points of the skeleton. The entire skeleton is then divided into several segments. Each segment corresponds to a branch in the ground truth. Concretely, TR is defined as

9 $$\begin{aligned} \textrm{TR} = \frac{\sum _{i=1}^{N} s_{i}\cdot \hat{p}_{i}}{\sum _{i=1}^{N} s_{i}}, \end{aligned}$$

where $s_{i}$ represents the value of the $i-$th voxel of skeleton ground truth and $\hat{p}_{i}$ represents the value of the $i-$th voxel of the binarized prediction map. BD is defined as

10 $$\begin{aligned} \textrm{BD} = \frac{\sum _{j=1}^{N_b} \lfloor \left( \sum _{i=1}^{N}\hat{p}_i \cdot b_i^j\right) / \sum _{i=1}^{N} b^j_i \rfloor }{N_b}, \end{aligned}$$

where $N_b$ represents the total number of the branches in the ground truth. $\hat{p}_i$ represents the value of the *i*-th voxel of the binarized prediction map and $b_i^j$ denotes the value of the *i*-th voxel of the *j*-th branch in the ground truth. $\lfloor \cdot \rfloor$ is the floor function. On the other hand, we utilized the precision rate, recall rate, and F-measure rate as evaluation metrics for abdominal artery segmentation. The three metrics are defined as

11 $$\begin{aligned} {\text {Precision}} = \frac{\text {TP}}{{\text {TP}} + {\text {FP}}}, \end{aligned}$$

12 $$\begin{aligned} {\text {Recall}} = \frac{\text {TP}}{{\text {TP}} + {\text {FN}}}, \end{aligned}$$

13 $$\begin{aligned} \mathrm{{F-measure}} = 2\cdot \frac{\mathrm{{Precision}} \cdot \mathrm{{Recall}}}{{\mathrm{{Precision}}} + \mathrm{{Recall}}}, \end{aligned}$$

where TP, FP, and FN represent the number of true positives, false positives, and false negatives, respectively. We repeated the training phase three times and reported the average and standard deviation values of each comparison method in our experiments.

**Table 2:**
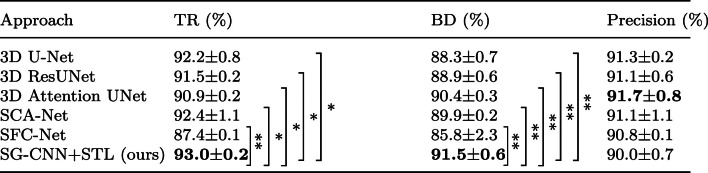
Evaluation of comparison methods over the testing set of the airway segmentation dataset

The bold fonts highlight the best performance terms. $$*: p$$ value $$\le 0.05$$, $$**: p$$ value $$\le 0.01$$, *ns* : *p* value $$> 0.05$$

### Implementation details

Our proposed deep model was implemented with PyTorch and processing was performed on a NVIDIA Tesla A100 GPU. We used stochastic gradient descent (SGD) as the optimization algorithm for both segmentation tasks. We initialized the learning rate at 0.0005, implementing a decay factor of 0.5 for every 20 epochs. The total epoch was set as 90. For the hyper-parameter tuning, we empirically set $\alpha =0.01$, $\beta =0.99$, and $\gamma =7$ for the skeleton structure segmentation.

### Evaluation on airway segmentation

**Fig. 5:**
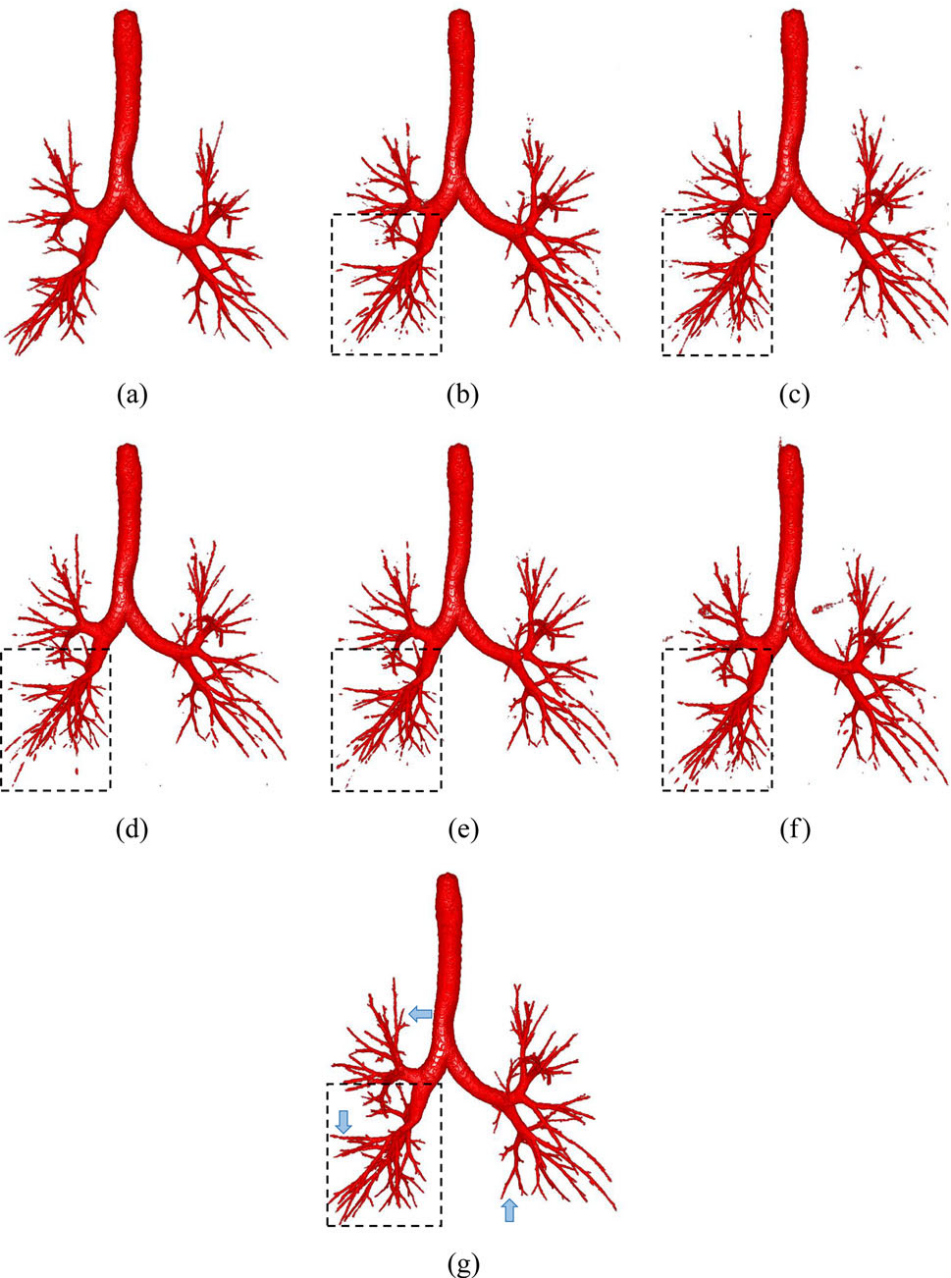
Airway segmentation results of comparison methods: **a** Ground truth, **b** 3D U-Net, **c** 3D residual U-Net, **d** 3D Attention U-Net, **e** SCA-Net, **f** SFC-Net, and **g** Our method. In the dotted boxes, our method has fewer broken segments and more segmented small branches, compared to other methods. The blue arrows point out several FPs in the predicted result of our method

We first evaluated our method of airway segmentation. Our method was compared with 3D U-Net [5], residual 3D U-Net [2], 3D Attention U-Net [14], skeleton context-aware fully convolutional network (SCA-Net) [15], and spatial fully connected network (SFC-Net) [1]. All the deep models of previous methods were trained using the Dice loss for tubular structure segmentation. We reproduced the comparison methods and evaluated their performance on the airway segmentation dataset. The quantitative results are shown in Table 2. Our method achieved the highest average TR and BD among the six compared deep models. The segmentation results of our deep model were TR: 93.0%, BD: 91.5%, and precision rate: 90.0%. The qualitative results are shown in Fig. 5. In addition, we conducted independent t tests to compare the performance of our method with other methods in terms of TR and BD. In the two metrics, our method outperformed other methods. Given an evaluation metric, the null hypothesis is that there is no significant difference between our method and the compared method in terms of the metric. We set the significance level at 0.05. The results (*p* values) are reported in Table 2.

### Evaluation on abdominal artery segmentation

**Fig. 6:**
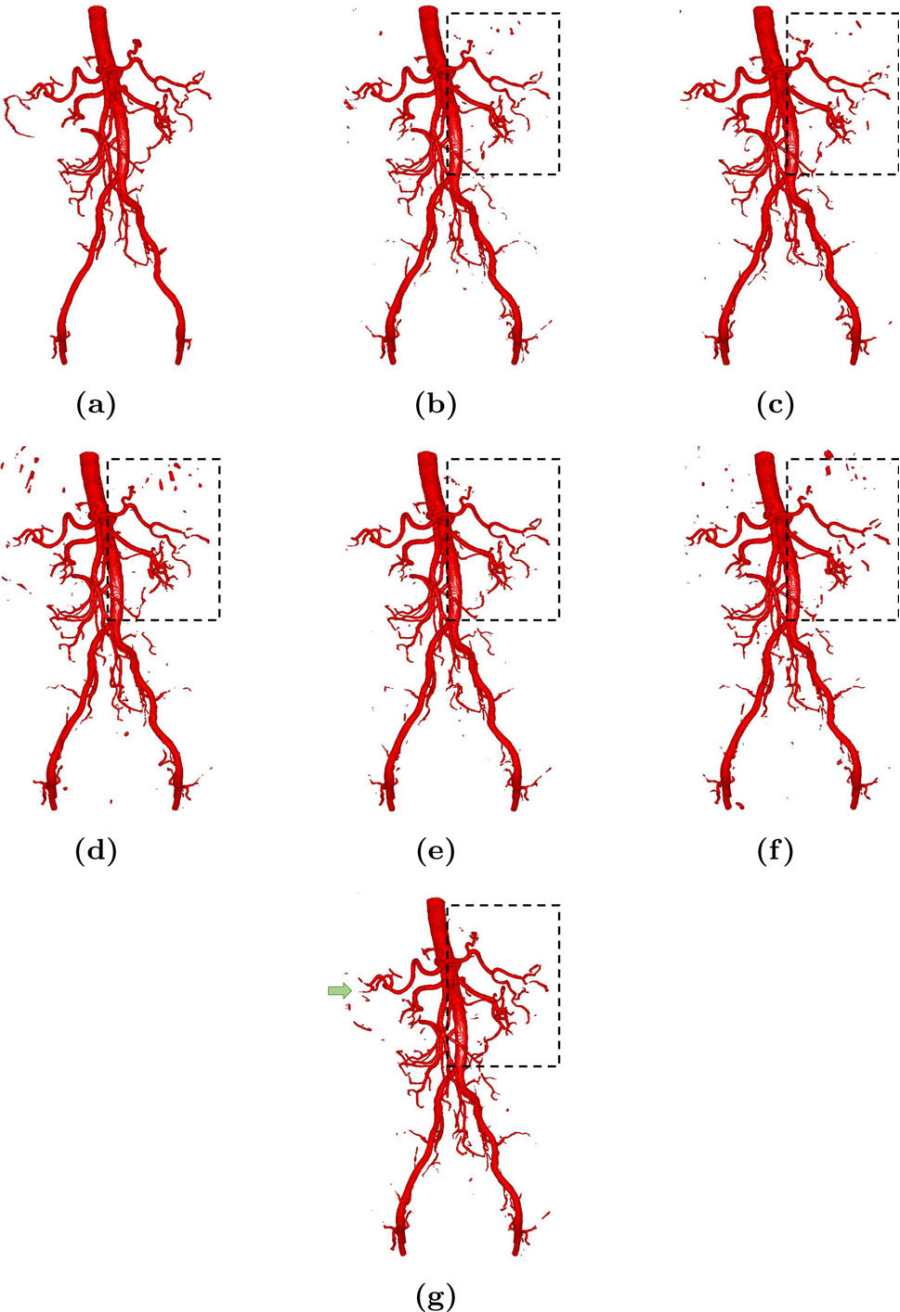
Abdominal artery segmentation results of comparison methods: **a** Ground truth, **b** 3D U-Net, **c** 3D residual U-Net, **d** 3D Attention U-Net, **e** SCA-Net, **f** SFC-Net, and **g** Our method. In the dotted boxes, our method well segment small arteries and preserve the connectivity of the arteries, outperforming other methods. The green arrow point out several FNs in the predicted result of our method

Subsequently, we evaluated our method on abdominal artery segmentation. Our method was compared with the same previous methods introduced in the airway segmentation evaluation. The quantitative results of the artery segmentation are shown in Table 3. Our method achieved a 97.7% precision rate and a 94.6% F-measure rate, outperforming comparison methods. The qualitative results are shown in Fig. 6. The independent t test results are shown in Table 3.

**Table 3:**
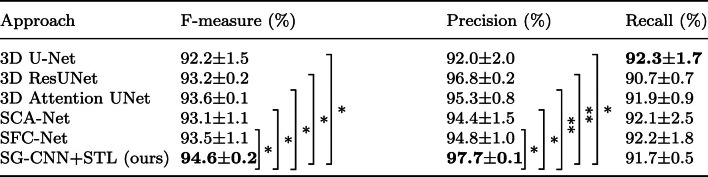
Evaluation of comparison methods over the testing set of the abdominal artery segmentation dataset. Independent t test results were also reported

The bold fonts highlight the best performance terms. $$*: p$$ value $$\le 0.05$$, $$**: p$$ value $$\le 0.01$$, *ns* : *p* value $$> 0.05$$

### Ablation study

We conducted an ablation study to verify the effectiveness of our method. The results are shown in Tables 4 and 5, corresponding to the airway and abdominal artery segmentation tasks, respectively. We evaluated our skeleton-guided stream and STL in the ablation study. The comparison objects are: (1) use our method without both the skeleton-guided stream and the STL (w/o SG), (2) replace STL with Dice loss (with Dice), and (3) replace STL with Tversky loss (with Tversky).

For the airway segmentation task, our method achieved the highest 93.0% TR and 91.5% BD among the three approaches. Our method with Tversky loss achieved the second-highest BD, and a competitive TR, among the three approaches. Our method with Dice loss underperformed other methods. For the abdominal artery segmentation task, our method outperformed other comparison methods with the highest 97.7% precision rate and 94.6% F-measure rate. Our method with Tversky loss achieved the second-highest recall rate and F-measure rate. Our method with Dice loss achieved the second-highest precision rate.

**Table 4:** Ablation study using the binary airway segmentation dataset

Approach	TR (%)	BD (%)	Precision (%)
Our method w/o SG	92.2 ± 0.8	88.3 ± 0.7	91.3 ± 0.2
Our method with Dice	83.9 ± 0.9	77.2 ± 0.3	**91.3 ± 0.1**
Our method with Tversky	92.1 ± 0.3	88.9 ± 0.2	91.0 ± 0.7
Our method	**93.0 ± 0.2**	**91.5 ± 0.6**	90.0 ± 0.7

The best performance of each evaluation term is highlighted using a bold font

**Table 5:** Ablation study using the abdominal artery segmentation dataset

Approach	Precision rate (%)	Recall rate (%)	F-measure (%)
Our method w/o SG	92.0 ± 2.0	92.3 ± 1.7	92.2 ± 1.5
Our method with Dice	95.6 ± 2.8	92.0 ± 1.1	93.7 ± 0.7
Our method with Tversky	94.8 ± 1.6	**93.0 ± 0.2**	93.8 ± 0.9
Our method	**97.7 ± 0.1**	91.7 ± 0.5	**94.6 ± 0.2**

The best performance of each evaluation term is highlighted using a bold font

## Discussion

Our fully automated method effectively extracted tubular structures from CT volumetric images.

### Performance of the proposed method

For airway segmentation, we compared our method to different deep models. The quantitative results given in Table 2 show that our method slightly improved segmentation performance in terms of the TR and BD (TR: 93.0%, BD: 91.5%, Precision rate: 90.0%), compared with previous methods. Many fully automated methods have been proposed for airway segmentation. Garcia-Uceda et al. [16] proposed a 3D U-Net-based method to extract airways. Their method achieved 70.3% TR over the EXACT’09 dataset. By contrast, our experiment used a larger dataset which is composed of the EXACT’09 and the LIDC dataset. Our method achieved a higher 93.3% TR among compared methods, indicating that more ground truth skeleton voxels were segmented in our prediction results. Qin et al. [17] employed an attention distillation mechanism in their deep model to improve segmentation accuracy. They used the same dataset as we used in our experiment. Their method achieved 96.2% BD and 90.7% TR. Although the BD of our method was slightly lower than that of Qin et al.’s method, our method performed a 93.3% TR, higher than that of Qin et al.’s method. It indicates that our method effectively segmented skeleton voxels of the ground truth in prediction maps. In Fig. 5, as emphasized in the dotted boxes, our method segmented more small branches, compared to previous methods. Meanwhile, we observed that our method exhibits a false positive issue, as depicted in Fig. 5, where several false positives were pointed out using blue arrows. Several branches which are not shown in ground truth were over-segmented. The false positives decrease the precision rate of our method. To address this, adjusting the weights of our loss functions is suggested as a potential solution. We can propose a trade-off hyper-parameter to balance the tubular structure segmentation loss function and the skeleton segmentation loss function, which should reduce the occurrence of false positives. Our method achieved competitive segmentation performance on the airway segmentation task.

**Fig. 7:**
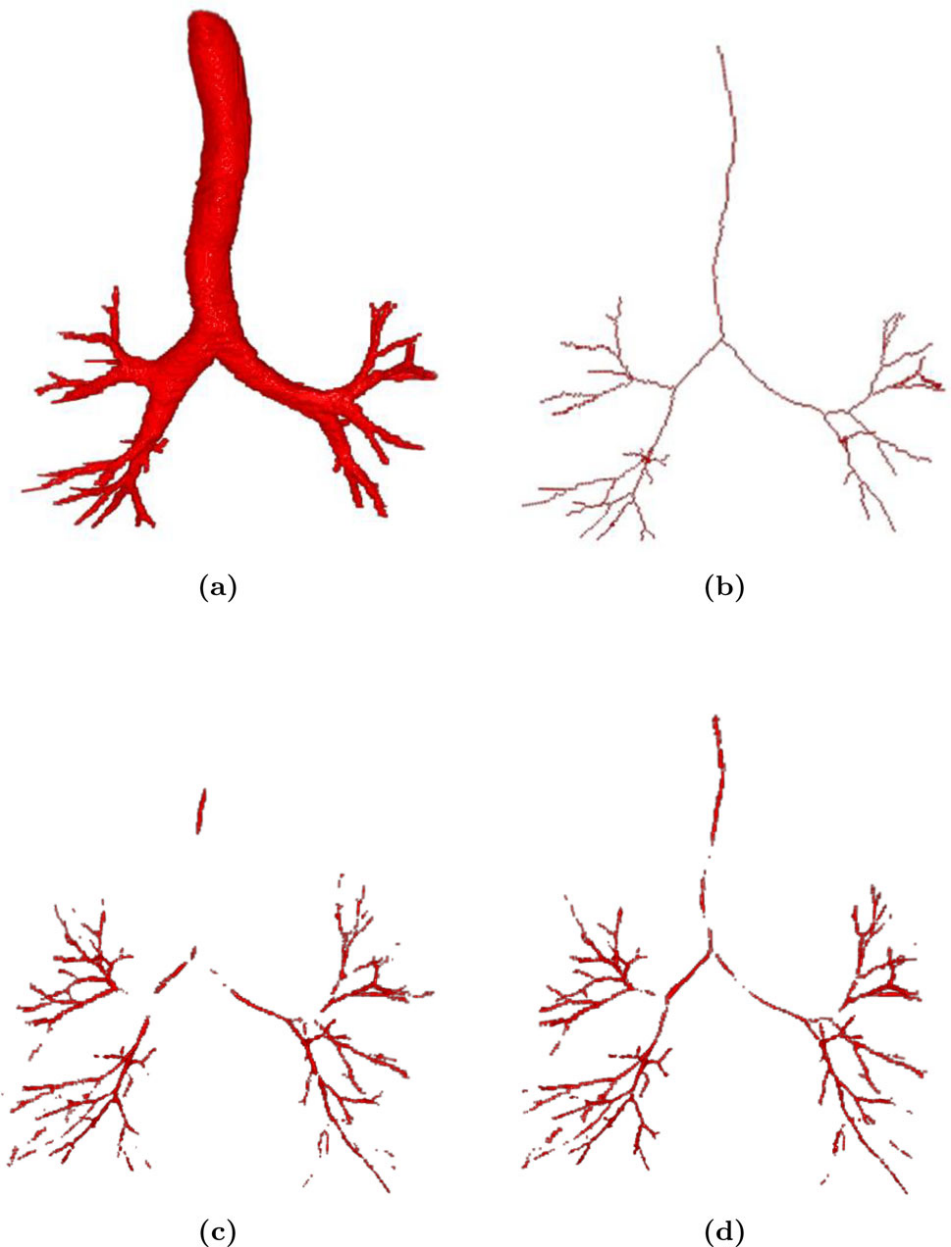
The example of a predicted skeleton map: **a** Ground truth, **b** Skeleton ground truth, **c** Predicted skeleton map using Tversky loss, **d** Predicted skeleton map using STL loss. Our STL loss provides more accurate predicted skeletons, especially the skeleton of trachea, compared to the Tversky loss

**Table 6:** Comparison on architectures using the binary airway segmentation dataset

Approach	TR (%)	BD (%)	Precision (%)
3D U-Net + STL	87.6 ± 0.2	85.2 ± 0.5	80.5 ± 0.7
3D ResUNet + STL	91.0 ± 0.7	88.8 ± 0.3	79.0 ± 1.1
3D Attention U-Net + STL	85.5 ± 0.5	83.5 ± 1.1	80.1 ± 0.9
SCA-Net + STL	87.2 ± 2.3	84.3 ± 1.9	80.1 ± 0.1
SFC-Net + STL	82.5 ± 1.3	81.0 ± 2.5	77.8 ± 0.7
SG-CNN + double STL	89.4 ± 0.8	86.6 ± 0.1	89.7 ± 2.4
SG-CNN+STL (ours)	**93.0 ± 0.2**	**91.5 ± 0.6**	**90.0 ± 0.7**

The best performance of each evaluation term is highlighted using a bold font

For the abdominal artery segmentation task, we also compared our method with several previous deep models. The quantitative results are given in Table 3. The 3D Attention U-Net slightly improved the F-measure rate, compared to other methods except ours. The results indicated that the attention mechanism is effective in tubular structure segmentation. Our method further achieved the highest overall segmentation performance (precision rate: 97.7%, recall rate: 91.7%, F-measure rate: 94.6%). In the qualitative results as shown in Fig. 6, our method performed well in segmenting small arteries (e.g., the dotted box area of prediction results). Oda et al. [18] proposed a 2.5D deep model for abdominal artery segmentation. Their method achieved 85.8% precision rate, 88.4% recall rate, and 87.1% F-measure rate. Our method improved the segmentation performance in terms of the precision rate, recall rate, and F-measure rate. In stead of using a 2.5D deep model, our 3D SG-CNN model could effectively leverage the information from tubular structure and skeleton feature maps, improving the segmentation performance. In conclusion, our method outperformed previous methods using both the airway dataset and the abdominal artery dataset. The skeleton information provided by the skeleton-guided stream effectively enables the main segmentation stream to segment tubular structures. Figure 6 shows that our method segmented more small abdominal arteries, compared to previous methods. However, the recall rate of our method was slightly lower than that of other methods, which might be caused by the increasing of false negatives, as pointed out by the green arrow in Fig. 6. The increase in false negatives could be influenced by the feature representation. Specifically, the structure of abdominal arteries is complex and hard to be represented in feature maps. Furthermore, our SG-CNN model leverages both the tubular structure feature maps and skeleton feature maps. In a skeleton feature map, the non-skeleton regions were treated as background regions. Although our method well segmented more small arteries by utilizing the skeleton information, the fusion of the skeleton feature maps might influence the feature representation of non-skeleton foreground regions in the tubular structure feature maps. We could adjust the weight of the two kinds of feature maps to emphasize the non-skeleton foreground regions, which should mitigate the issue. Different segmentation tasks validate the robustness of our method.

### Effectiveness of the proposed method

We conducted an ablation study using both the airway segmentation dataset and the abdominal artery segmentation dataset. The quantitative results are presented in Tables 4 and 5. In the airway segmentation ablation study, our method with Dice loss did not outperform our method without SG-CNN. It indicated that the Dice loss might not be feasible for airway segmentation in our method. Our method with Tversky loss improved the segmentation performance in terms of TR and BD, compared to our method without the SG module. Our method with STL further increased the TR and BD, compared to the Dice loss and the Tversky loss. Similarly, in the abdominal artery segmentation ablation study, our method with STL outperformed other three comparison methods. Our STL differs from the Tversky loss by incorporating element-wise gradient ratio adaptation. The STL could enhance the skeleton segmentation performance, which means that the skeleton feature map could provide sufficient skeleton information for the main tubular structure segmentation stream, improving the segmentation performance of tubular structures. The results indicated that our STL plays a crucial role in leading to improved true positive regions.

We also conducted independent t test to evaluate the effectiveness of our method. For the airway segmentation task, there is significant difference between our method and previous methods in terms of the TR and the BD. In the additional evaluation on the independent t test, there is significant difference between our method and the attention U-Net in terms of the precision rate. Similarly, in the abdominal artery segmentation task, there is significant difference between our method and previous methods in terms of the F-measure and the precision rate. Although there is no significant difference between the recall rates of the 3D U-Net and that of our method, the difference between the average recall rate of our method and that of the 3D U-Net was very small. (i.e., the difference is only 0.6%). In addition, the precision rate and the F-measure of our method outperformed those of previous methods, with significant difference. The overall results validates the effectiveness of our method.

We further evaluated the skeleton prediction performance of our skeleton-guided stream. The qualitative results of predicted skeleton maps are shown in Fig. 7. Due to the poor performance on skeleton prediction, we did not report the qualitative results of the Dice loss. We observed that the predicted skeleton map using our STL loss outperformed the skeleton map using the Tversky loss. In addition, the predicted skeleton map well represented structure information of the tubular structure. The results validated the effectiveness of skeleton information in our method.

### Limitations

Our proposed loss function, STL, is specifically designed to improve the segmentation accuracy of low confidence regions characterized by prediction probability close to 0.5, improving the segmentation performance on the hard-to-segment regions. For the limitations of our method, our proposed loss may not appropriately adapt the gradient ratio for regions with high confidence regions, which could affect the segmentation performance in those areas. We conducted additional experiments which compare our SG-CNN architecture with other deep models using the airway segmentation data set, as shown in Table 6. All of the deep models were trained with our STL. We observed that applying STL to deep models decrease the segmentation performance in terms of TR, BD, and precision rate. Nevertheless, our method with double STL still achieved competitive segmentation accuracy, compared to previous methods with our STL, showing the effectiveness of our SG-CNN architecture.

## Conclusion

In this paper, we present a fully automated method for the segmentation of 3D tubular structures from CT volumetric images. We propose a novel 3D convolutional network to leverage the skeleton information of tubular structures. Moreover, we present a sigmoid-adaptive Tversky loss for a skeleton-guided task. Experimental results showed that our method provided competitive results compared to previous methods.

The skeleton information has been shown to be effective in tubular structure segmentation. We will explore the applications of skeleton information in different deep models. Moreover, our proposed loss might not perform well in easy-to-segment regions. We will further investigate the issue and modify our proposed loss to improve the segmentation performance.
